# Pharmacological characterization of cnidarian extracts from the Caribbean Sea: evaluation of anti-snake venom and antitumor properties

**DOI:** 10.1186/s40409-018-0161-z

**Published:** 2018-08-28

**Authors:** Cláudia S. Oliveira, Cleópatra A. S. Caldeira, Rafaela Diniz-Sousa, Dolores L. Romero, Silvana Marcussi, Laura A. Moura, André L. Fuly, Cicília de Carvalho, Walter L. G. Cavalcante, Márcia Gallacci, Maeli Dal Pai, Juliana P. Zuliani, Leonardo A. Calderon, Andreimar M. Soares

**Affiliations:** 1 Centro de Estudos de Biomoléculas Aplicadas a Saúde (CEBio), Fundação Oswaldo Cruz de Rondônia (Fiocruz Rondônia), Porto Velho, RO Brazil; 2 Brazilian Marine Biotechnology Network (BioTecMar Network), Porto Velho, Brazil; 3 Departamento de Medicina, Universidade Federal de Rondônia (UNIR), Porto Velho, RO Brazil; 4 Centro de Estudios de Proteínas, Facultad de Biología, Universidad de La Habana, Havana, Cuba; 5 Departamento de Química, Universidade Federal de Lavras (UFLA), Lavras, MG Brazil; 6 Departamento de Biologia Celular e Molecular (GCM), Instituto de Biologia, Universidade Federal Fluminense (UFF), Niterói, RJ Brazil; 7 Departamento de Farmacologia, Instituto de Biociências, Universidade Estadual Paulista (UNESP), Botucatu, SP Brazil; 8 Instituto de Ciências Biológicas, Departamento de Farmacologia, Universidade Federal de Minas Gerais (UFMG), Belo Horizonte, MG Brazil; 9 Departamento de Morfologia, Instituto de Biociências, Universidade Estadual Paulista (UNESP), Botucatu, SP Brazil; 10 Centro Universitário São Lucas (UniSL), Porto Velho, RO Brazil

**Keywords:** Caribbean sea cnidarians, Bioprospection, Antiophidic, Antitumor, Natural products

## Abstract

**Background:**

Cnidarians produce toxins, which are composed of different polypeptides that induce pharmacological effects of biotechnological interest, such as antitumor, antiophidic and anti-clotting activities. This study aimed to evaluate toxicological activities and potential as antitumor and antiophidic agents contained in total extracts from five cnidarians: *Millepora alcicornis*, *Stichodactyla helianthus, Plexaura homomalla, Bartholomea annulata* and *Condylactis gigantea* (total and body wall).

**Methods:**

The cnidarian extracts were evaluated by electrophoresis and for their phospholipase, proteolytic, hemorrhagic, coagulant, fibrinogenolytic, neuromuscular blocking, muscle-damaging, edema-inducing and cytotoxic activities.

**Results:**

All cnidarian extracts showed indirect hemolytic activity, but only *S. helianthus* induced direct hemolysis and neurotoxic effect. However, the hydrolysis of NBD-PC, a PLA_2_ substrate, was presented only by the *C. gigantea* (body wall) and *S. helianthus*. The extracts from *P. homomalla* and *S. helianthus* induced edema, while only *C. gigantea* and *S. helianthus* showed intensified myotoxic activity. The proteolytic activity upon casein and fibrinogen was presented mainly by *B. annulata* extract and all were unable to induce hemorrhage or fibrinogen coagulation. Cnidarian extracts were able to neutralize clotting induced by *Bothrops jararacussu* snake venom, except *M. alcicornis*. All cnidarian extracts were able to inhibit hemorrhagic activity induced by *Bothrops moojeni* venom. Only the *C. gigantea* (body wall) inhibited thrombin-induced coagulation. All cnidarian extracts showed antitumor effect against Jurkat cells, of which *C. gigantea* (body wall) and *S. helianthus* were the most active; however, only *C. gigantea* (body wall) and *M. alcicornis* were active against B16F10 cells.

**Conclusion:**

The cnidarian extracts analyzed showed relevant in vitro inhibitory potential over the activities induced by Bothrops venoms; these results may contribute to elucidate the possible mechanisms of interaction between cnidarian extracts and snake venoms.

## Background

Marine organisms, which comprise half of the total global biodiversity, have been recognized as the largest remaining reservoir of novel compounds to be evaluated for drug activity [1–6]. Animals belonging the phylum Cnidaria are of great importance for studies of pharmacological and toxicological assessments. The composition of cnidarian venoms remains incompletely elucidated. However, several of their compounds have been described, including peptides, proteins, purines, quaternary ammonium compounds, biogenic amines and betaines [1, 7–11].

Venoms from such animals as snakes [12–14], scorpions [15–18], anurans [19, 20], cone snails [21, 22] and cnidarians [23–25] have been used as a source of bioactive compounds for the prospection of lead compounds potentially useful for the development of new anticancer therapies [26, 27]. This fact has provoked a growing worldwide interest in the screening of proteins, peptides, marine natural products (MNPs) from cnidarians in order to discover new anticancer bioactive compounds [28, 29].

The use of genomic and proteomic approaches had permitted a rapid increase in the number of sequences from cnidarians deposited in protein and gene databases [30–32]. Some of these toxins have been used for the development of anticancer molecules. One interesting example is the hemolytic toxin (HT) from *Stichodactyla helianthus* sea anemone which was conjugated with an antibody towards an antigen expressed on immature T lymphocytes (IOR-T6) producing an o-hemolytic hybrid IOR-T6-HT that showed toxicity against CEM cells expressing the IOR-T6 antigen and non-toxic effects for K562 cells without the antigen [33].

Additionally, several marine natural products are able to inhibit the toxic effects of snake venoms, such as extracts from *Plocamium braziliense* [34], *Canistrocarpus cervicornis* [35] and seaweed *Prasiola crispa* [36]. The marine extracts that also inhibit PLA_2_ activity include manoalide [37], vidalols, and a group of terpenoids that contain masked 1,4-dicarbonyl moieties. Furthermore, the biotechnological potential of PLA_2_ inhibitors may provide therapeutic molecular models that exert antiophidian activity to supplement the conventional serum therapy against these multifunctional enzymes [38, 39].

This study aimed to evaluate toxicological activities and their efficacy against tumor and snake-venom toxic activities from five Caribbean Sea cnidarian species of the hydrozoa class: *Millepora alcicornis, Plexaura homomalla* and Cnidarians of the anthozoa class: *Condylactis gigantea* (total and body wall), *Stichodactyla helianthus,* and *Bartholomea annulata*.

## Methods

### Materials and reagents

The synthetic fluorescent substrates Acyl 6:0 NBD phospholipids, NBD-phosphatidylcholine (PC) and NBD-phosphatidic acid (PA) were purchased from Avanti Polar Lipids Inc. (USA). The reagents used in the electrophoresis, salts and other reagents were obtained from Sigma Chemical Company (USA).

### Cnidarian extracts

The cnidarians specimens were collected in the coast of Havana City during a one-year period. The extracts of corals were obtained as previously described by [40], whereas anemone extracts were obtained according to [41]. Protein quantitation was based on the Bradford method (BioRad) using bovine serum albumin (BSA) as a standard.

### Animals

Adult male mice weighing 25 to 30 g were maintained under a 12 h light-dark cycle (lights on at 07:00 h) in a temperature-controlled environment (22 ± 2 °C) for at least ten days prior to the experiments. Food and water were freely available. Animal procedures were in accordance with the guidelines prepared by the Committee on Care and Use of Laboratory Animal Resources, National Research Council, USA. The ethical aspects related to the project were approved by the Ethics Committee on Animal Use (No. 2012/1) and the Ethics Committee (102/2009) for Research on Human Beings from Brazil (CAAE: 14204413.5.0000.0011).

### Electrophoresis

SDS-PAGE 12.5% (m/v) was carried out as previously described [42]. 500 μg samples *C. gigantea* (body-wall), *C. gigantea* (total), *M. alcicornes*, *S. helianthus*, *P. homomalla* and *B. annulata* were pretreated in reducing conditions (SDS plus β-mercaptoethanol) at 100 °C for 5 min. Gels were stained with 0.1% Coomassie brilliant blue R-350 in ethanol: acetic acid (5:1, *v*/v) for 15 min and discolored in 10% acetic acid. The molecular mass was estimated by interpolation from a linear logarithmic plot of relative molecular mass versus distance of migration using standard molar mass markers (SDS7 Sigma-Aldrich).

### Phospholipase activity

The Phospholipase A_2_ (PLA_2_) activity was measured using the indirect hemolytic assay on agarose gels containing red blood cells and egg yolk phospholipids [43]. The hemolytic activity was evaluated spectrophotometrically using suspensions of fresh human RBC (red blood cells) as previously described [44, 45].

PLA_2_ activity was evaluated also through the hydrolysis of synthetic fluorescent phospholipid, using the fluorescent substrate Acyl 6:0 NBD phospholipid, NBD-phosphatidylcholine (NBD-PC). The assay was performed using a spectrofluorimeter (Shimadzu, RF-5301PC, software RFPC) with excitation and emission wavelengths of 460 and 534 nm, respectively. The enzymatic activity of each cnidarian extract was evaluated for 250 s after the addition of substrate (3.3 μg/mL, final concentration) in a reaction medium containing 50 mM Tris-HCl, and 8 mM CaCl_2_, pH 7.5, at room temperature.

### Proteolytic activity assay

Proteolytic activity upon fibrinogen was measured as described by [46] with some modifications. Fibrinogen (70 μg) diluted in PBS was incubated with different amounts of cnidarian extracts diluted in 20 μL buffer (pH 7.5) at 37 °C for 2 h. The reaction was stopped with 20 μL of a solution containing 10% (v/v) glycerol, 10% (v/v) β-mercaptoethanol, 2% (v/v) SDS, and 0.05% (w/v) bromophenol blue. Fibrinogen hydrolysis was demonstrated by SDS-PAGE using 12% polyacrylamide gels. Proteolytic activity upon casein was measured as described by [47]. Cnidarian extracts (100 and 500 μg) were incubated for 30 min at 37 °C in a solution of 0.1 M Tris-HCl pH 9.0 containing 1% casein. After the incubation period, 1.5 mL of 30% TCA was added to each sample to stop the enzymatic reaction and centrifuged at 340 *x g* for 25 min. Then, the samples were read on a spectrophotometer at a wavelength of 280 nm. One unit of protease activity was defined as the amount of enzyme that produces an increase in absorbance of 0.001 units/minute at 280 nm.

### Hemorrhagic activity assay

Hemorrhagic activity was quantitatively estimated by the method of [48] with some modifications. Groups of six Swiss mice (18-22 g) were shaved on the back and then intradermally (i.d.) injected with different doses of cnidarians extracts or snake venoms, in 50 μL of phosphate buffered saline (PBS). After 2 h, animals were anesthetized and euthanized. The shaved back skin was removed and the hemorrhagic halo diameter was measured. The minimum hemorrhagic dose (MHD) was obtained from the mean of these diameters (mm). The MHD is defined as the dose of snake venom or extract that produces a hemorrhagic lesion of 10 mm diameter after 2 h.

### Coagulant activity assay

The clotting time was determined by mixing 20 μL of the samples (in 0.15 M NaCl, pH 7.4) with 200 μL of citrated bovine plasma at 37 °C. The *B. jararacussu* snake venom (20 μg) was assayed in order to determine the minimum coagulant dose (corresponding to the time between 1 and 1.2 s – 100% activity). For the neutralization trials, the snake venom was previously incubated with different cnidarian extracts for 30 min at 37 °C, at different proportions (1:5, 1:10 and 1:30, w/w).

### Neuromuscular blocking

Mice were euthanized by exsanguination after previous cervical dislocation. Phrenic-diaphragm (PD) preparation was removed and mounted vertically in a conventional isolated organ-bath chamber containing 15 mL of physiological solution of the following composition (mmol/L): NaCl, 135; KCl, 5; MgCl_2_, 1; CaCl_2_, 2; NaHCO_3_, 15; Na_2_HPO_4_, 1; glucose, 11. This solution was bubbled with carbogen (95% O_2_ and 5% CO_2_). The preparation was attached to an isometric force transducer (Grass, FT03) for recording the twitch tension. The transducer signal output was amplified and recorded on a computer via a transducer signal conditioner (Gould, 13–6615-50) with an Acquire Lab Data Acquisition System (Gould). The resting tension was 5 g; indirect contractions were stimulated by supramaximal pulses (0.2 Hz, 0.5 ms) delivered from an electronic stimulator (Grass-S88 K) and applied to the phrenic nerve by means of a suction electrode. The preparation was allowed to stabilize for 45 min before the addition of a single concentration of toxin [49].

### Muscle-damaging activity

#### Morphological analysis

At the end of the myographic study, the diaphragm muscle was removed from the bath and frozen in liquid nitrogen. Transverse sections (8 mm thick) were cut out at − 20 °C in a cryostat and stained with hematoxylin and eosin (HE) prior to examination by light microscopy [50]. Muscle damage was quantified in HE stained preparations, using an Analysis Imaging System (Leica, Qwin). The number of fibers with lesions was expressed as a percentage of the total number of cells (muscle damage index), in three non-overlapping non-adjacent areas of each muscle, observed at the same magnification.

#### Creatine kinase release

The creatine kinase (CK) assay was carried out using the CK-UV kinetic kit from *Sigma Chem. Co.* Different cnidarian extracts were injected (i.m., 50 μL) into Swiss male mice weighing 18–22 g (*n* = 6). The control animals received 0.15 M PBS. After 3 h, the blood from the tail was collected in heparin-coated tubes and centrifuged for plasma separation. The amount of CK was then determined using 4 μL of plasma, which was incubated for 3 min at 37 °C with 1.0 mL of the reagent. Enzyme activity was expressed in international units per liter (IU/L), with one unit of activity corresponding to phosphorylation of 1 μmol of creatine/min at 25 °C.

### Edema inducing activity

Groups of six Swiss male mice (18–22 g) were injected in the sub plantar region with different doses of cnidarian extracts in 50 μL of PBS. After 0.5, 1 and 3 h, the paw edema was measured using a low-pressure spring caliper (Mytutoyo-japan) [51, 52]. The zero time values were then subtracted and the differences reported as median % ± S.D.

### Cytotoxic activity

Tumor cell cytotoxic activity of cnidarian extracts on human acute T-cell leukemia (Jurkat) and B16F10 cell lines were assayed using the MTT method according to [53]. Cells were dispersed in 96-well plates at a density of 1 × 10^5^ cells per well. After 24 h of culture, the media were removed and fresh media, with or without different concentrations of samples, were added into the wells and incubated for 24 h. The extracts were evaluated at 1000, 100 and 10 μg/mL concentrations using Vincristine as positive control (100 μg/mL). Results were expressed as a percentage (%).

### Statistical analysis

Results were expressed as mean ± S.D. Data was analyzed by ANOVA complemented by the Tukey-Kramer test, using the statistical program GraphPad 5.0. Values of *p* < 0.05 were considered significant.

## Results and discussion

**Fig. 1:**
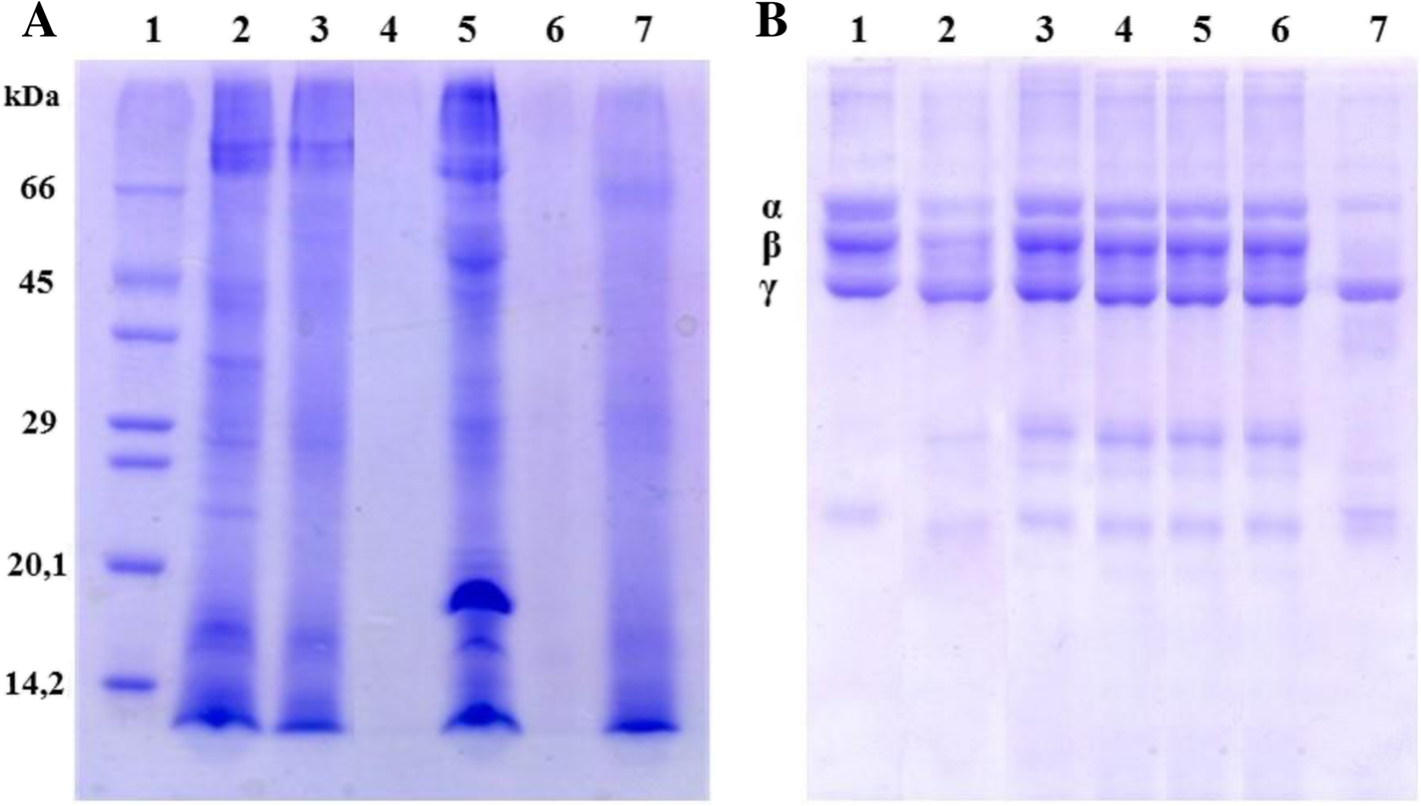
(**a**) PAGE in the presence of SDS and β-mercaptoethanol. Lanes: 1 – standard, molecular-weight markers; 2 – *C. gigantea* (body-wall); 3 – *C. gigantea* (total); 4 – *M. alcicornes*; 5 – *S. helianthus*; 6 – *P. homomalla*; 7 – *B. annulata*; samples were applied containing 500 μg of each extract. (**b**) Fibrinogenolytic activity of cnidarian extracts.1 – Fibrinogen; 2 – fibrinogen + *C. gigantean* (body-wall); 3 – fibrinogen + *C. gigantea* (total); 4 – fibrinogen + *M. alcicornes*; 5 – fibrinogen + *S. helianthus*; 6 – fibrinogen + *P. homomalla;* 7 – fibrinogen + *B. annulata.* Fibrinogen hydrolysis was demonstrated by SDS-PAGE using 12% polyacrylamide gels

The SDS-PAGE analysis in denaturing conditions of the cnidarian extracts showed the difference between extracts of *C. gigantea* (body-wall), *C. gigantea* (total), *S. helianthus, B. annulata*, *M. alcicornes* and *P. homomalla*. Considering that the extracts were obtained from the entire organism, it should be noted that the anatomy of *M. alcicornes* and *P. homomalla* is different from that of anemones. For this reason, the method of protein extraction must be differentiated for these organisms. Thus, it is possible that in the 500 μg extract applied to the electrophoresis, a low protein yield made it impossible to visualize bands on the polyacrylamide gel (Fig. 1a).

**Fig. 2:**
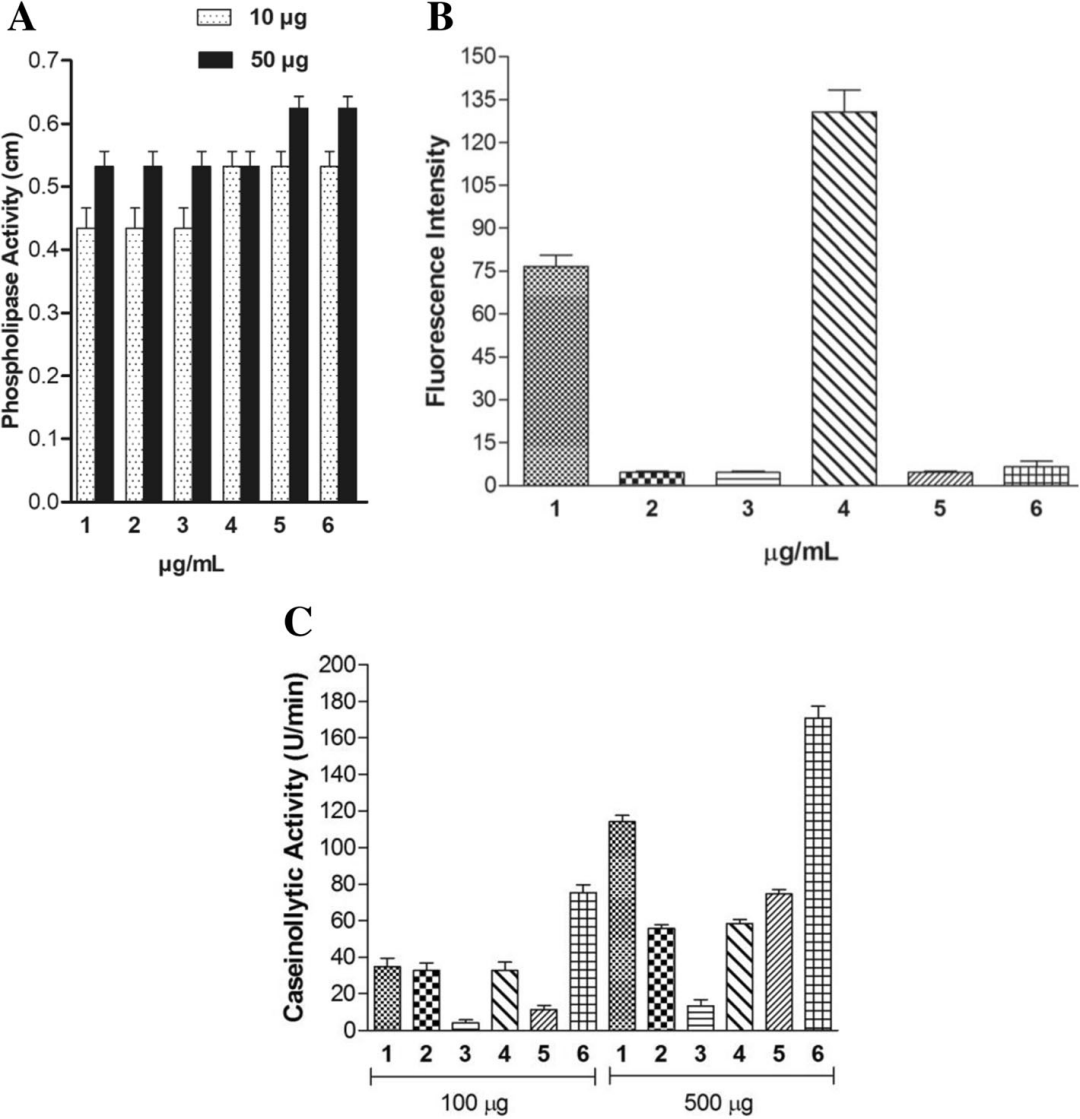
Phospholipase and proteolytic activities induced by cnidarian extracts. (1) *C. gigantea* (body-wall), (2) *C. gigantea* (total), 3) *M. alcicornis,* (4) *S. helianthus* (5) *P. homomalla* and (6) *B. annulata.* (**a**) The PLA_2_ activity using the indirect hemolytic assay on agarose gels containing red blood cells and egg-yolk phospholipids and by (**b**) Hydrolysis of the NBD-PC by cnidarian extracts (1–6). (**c**) Proteolytic activity upon 1% casein evaluated with 100 and 500 μg of cnidarian extracts for 30 min at 37 °C. Results are reported as mean % ± SD (*n* = 3)

The extracts of *S. helianthus* and *C. gigantea* (body-wall) hydrolyzed the NBD-PC substrate that is specific for the PLA_2_ enzymes, which was not observed for the other extracts tested (Fig. 2b). Martins and coworkers [54] isolated a PLA_2_ (CgPLA2) composed of 14 kDa dimers and 29 kDa monomer from the *C. gigantea* extract. Another study reported showed that Sticholysin I and II, two 19 kDa pore-forming cytokines, present in the *S. helianthus* extract, low activity against specific substrates for phospholipase activity when tested alone [55].

However, fractions enriched with these two molecules together demonstrated a significant increase in phospholipase activity [56]. Although CgPLA_2_ and Sticholysin I and II are known to exert phospholipase activity, it should be emphasized that the experiments were carried out with total extract. It is possible that these molecules are responsible for the hydrolysis promoted against the NBD-PC substrate, but we do not rule out the existence of other molecules that are components of the extract, which alone or in clusters may be acting in the hydrolysis of the NBD-PC substrate.

**Fig. 3:**
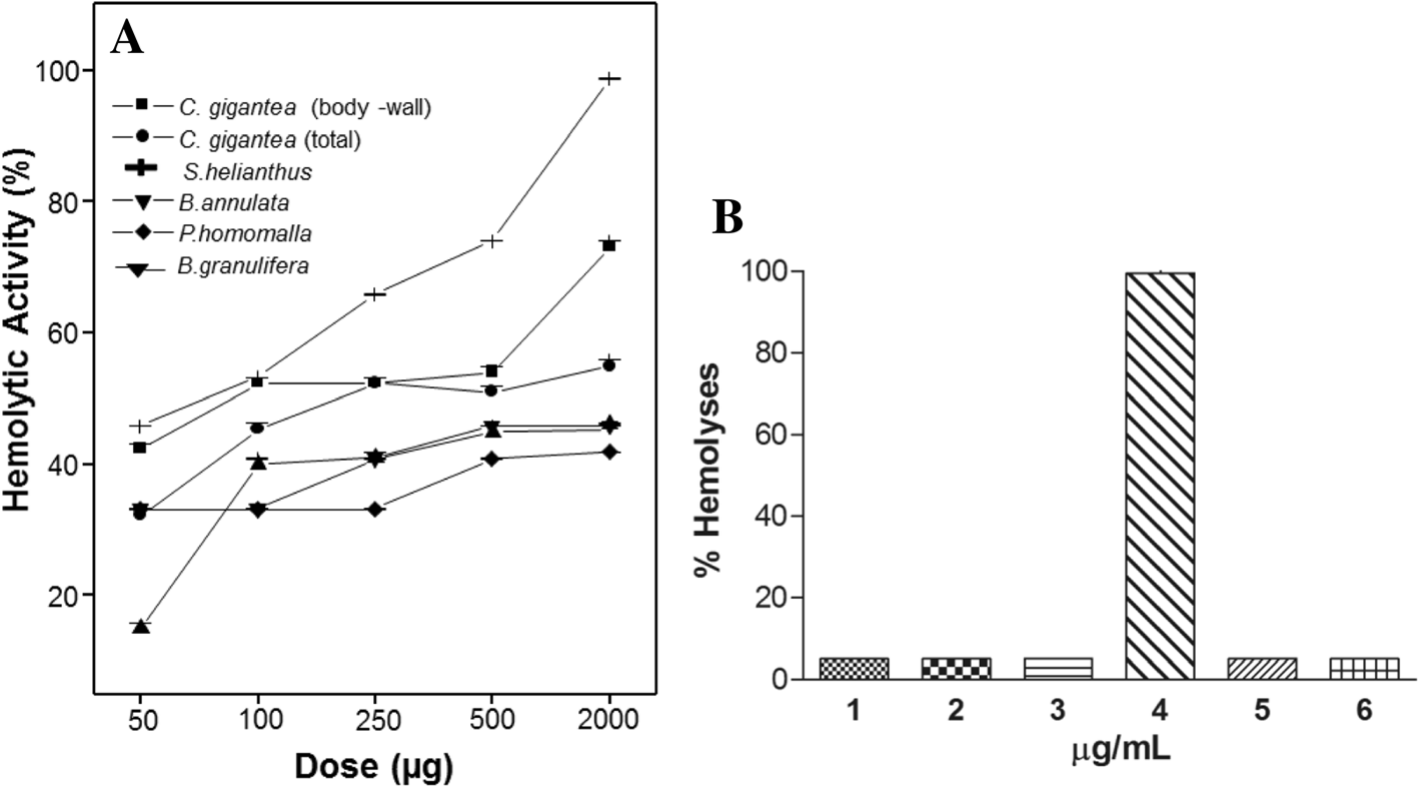
Hemolytic activity of cnidarian extracts. (1) *C. gigantea* (body-wall), (2) *C. gigantea* (total), (3) *M. alcicornis,* (4) *S. helianthus,* (5) *P. homomalla* and (6) *B. annulata.* (**a**) Percentage of hemolytic activity tested at different doses from 50 to 2000 μg; (**b**) the hemolysis was tested at the same 200 μg/mL concentration

As shown in Fig. 2a, all extracts displayed indirect hemolytic activity. However, only extracts of *S. helianthus* and *C. gigantea* showed direct hemolytic activity by lysis of red blood cells, in a concentration-dependent manner (Fig. 3a, b). Hemolysis was provoked not only by anemone extract as observed in *B. annulate* [57] and *S. helianthus* [58] but also by *M. alcicornes* aqueous extract [8]. In addition, Sticholysin II toxin isolated from *S. helianthus* has also been described for its hemolytic capacity [45, 59–63].

The extracts of *C. gigantea* (body wall) and *B. annulata* were able to partially hydrolyze the α and β chains of fibrinogen; however, *S. helianthus, M. alcicornis* and *P. homomalla* were incapable of hydrolyze fibrinogen efficiently (Fig. 1b). The fibrinogenolytic assay was carried out using 50 μg of the cnidarian extracts, whose proteolytic activity upon casein was evaluated; furthermore, the extract of *B. annulata* (100 μg) hydrolyzed casein at 80 U/min. The extracts of *C. gigantea* (body wall) and *B. annulata* at 500 μg hydrolyzed casein at 118 and 170 U/min, respectively (Fig. 2c).

**Fig. 4:**
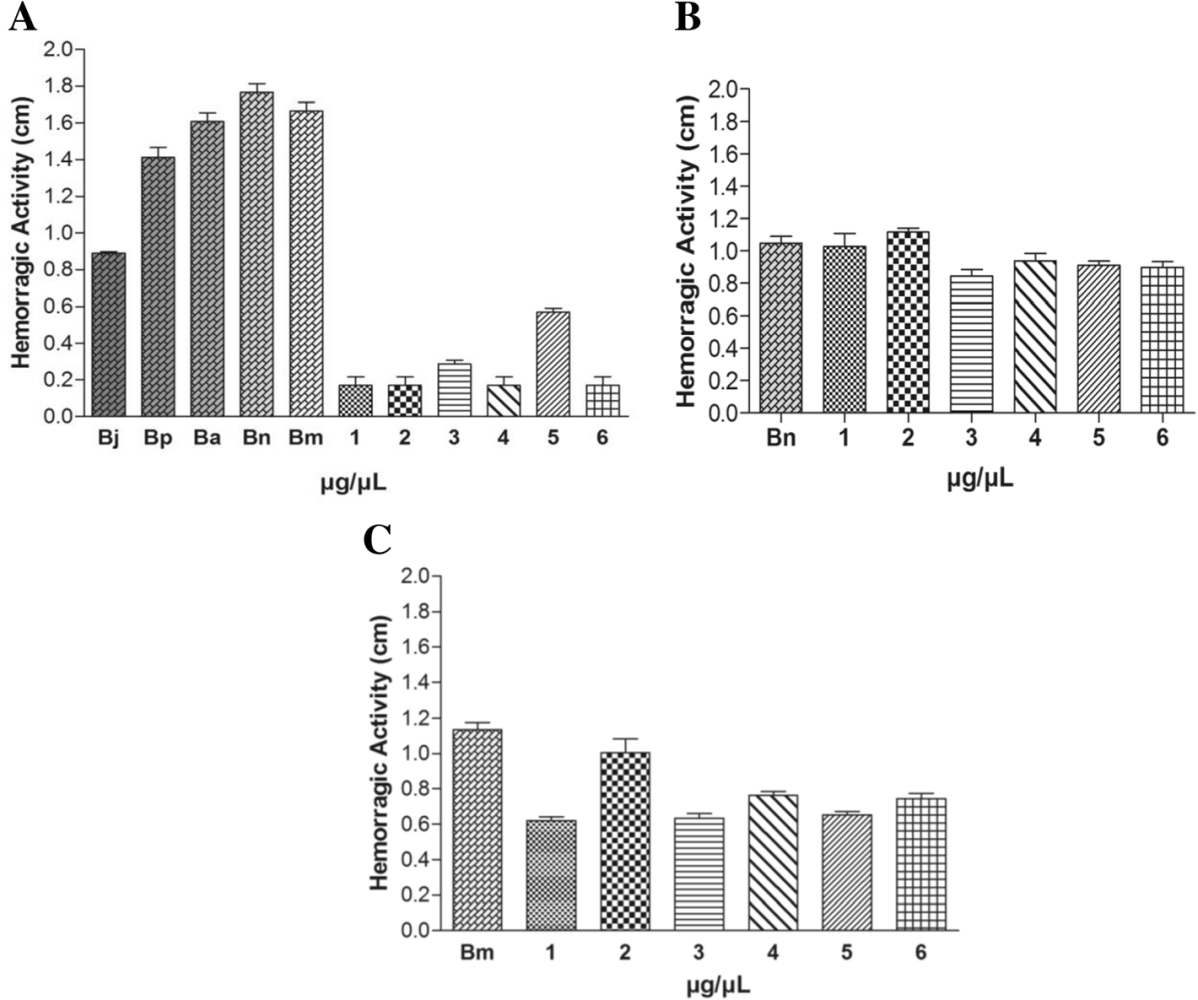
Induction and Inhibition of hemorrhagic activity. (**a**) Hemorrhage induced by snake venoms: (Bj) *B. jararacussu*, (Bp) *B. pirajai*, (Ba) *B. alternatus*, (Bn) *B. neuwiedi*, (Bm) *B. moojeni* - all venoms at 30 μg and, cnidarian extracts: (1) *C. gigantea* (body-wall), (2) *C. gigantea* (total), (3) *M. alcicornis,* (4) *S. helianthus* (5) *P. homomalla*, (6) *B. annulata -* all extracts at 150 μg. (**b**) Inhibition of hemorrhagic activity induced by *B. neuwiedi* (Bn) at 20 μg and; (**c**) Inhibition of haemorrhagic activity induced by *B. moojeni* (Bm) at 10 μg. Results are reported as mean % ± S. D (n = 3)

As to the hemorrhagic effect, extracts of *M. alcicornis* and *P. homomalla* induced bleeding at a concentration of 150 μg (Fig.4a) and inhibited bleeding induced by *Bothrops* snake venom. All cnidarians extracts tested inhibited the hemorrhagic activity induced by *B. moojeni* venom at a ratio of 1:30 w/w, showing approximately 40% inhibition in the presence of extracts of *C. gigantea* (body wall), *P. homomalla* and *M. alcicornis* (Fig.4c). Interestingly, the *B. neuwiedi* venom extracts did not inhibit hemorrhage (Fig. 4b).

**Fig. 5:**
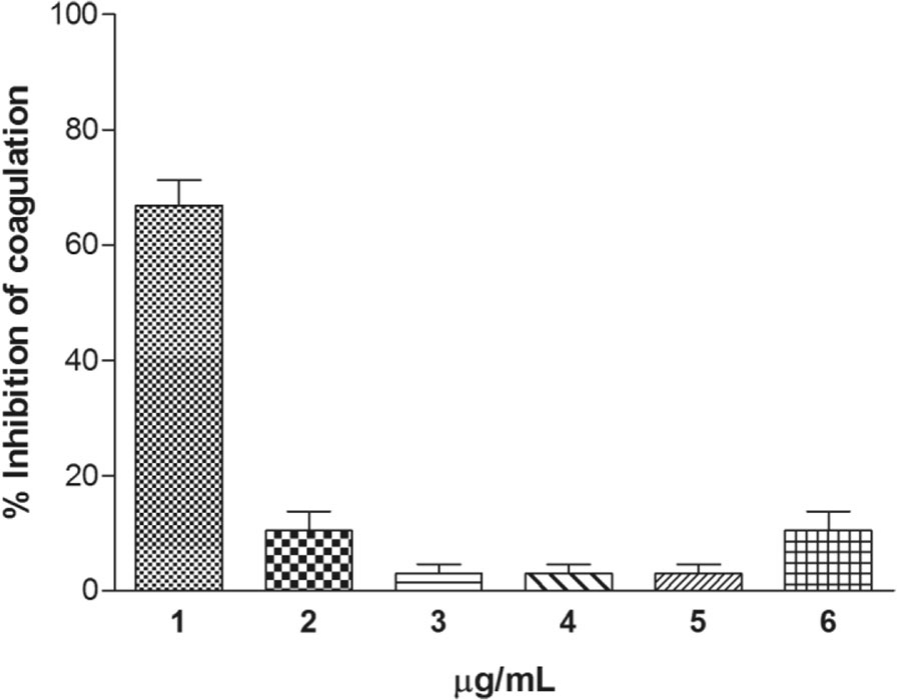
Effect of cnidarian extracts on coagulation.(1) *C. gigantea* (body-wall), (2) *C. gigantea* (total), (3) *M. alcicornis,* (4) *S. helianthus,* (5) *P. homomalla*, (6) *B. annulata,* 200 μg/mL of the extract were pre-incubated with fibrinogen; clotting was then started by the addition of thrombin and monitored at A_405_ nm. Results are reported as mean % ± S. D (n = 3)

Coagulant activity was not induced by cnidarian extracts. However, all cnidarian extracts except *M. alcicornis* inhibited the coagulant activity induced by *B. jararacussu* venom at the ratios of 1:5 and 1:10 w/w. In this assay, the *B. annulata* extract at a 1:30 w/w ratio showed the greatest ability to delay the clotting time of citrated plasma after the addition of *B. jararacussu* venom for more than 40 min (Table 1). The other extracts tested at the 1:30 w/w ratio presented lower inhibitory effect (Fig. 5). Additionally, *C. gigantea* body-wall extract at a concentration of 200 μg/mL were able to inhibit thrombin-induced coagulation. This activity can be produced by the presence of protease inhibitors, as reported by [64], who showed the presence of protease inhibitory activity in the extract from the marine sponge *Xetospongia muta* (Poriphera) and in the sea anemones *B. granulifera* and *B. annulata*. According to these authors, this activity was dose-dependent and the molecule responsible for the inhibition had a low molecular weight. Di Bari and coworkers [65] also described the presence of protease inhibitors in aqueous extracts of marine sponges; all extracts were able to inhibit the activity and expression of matrix metalloproteinases (MMP-2 and MMP-9) in mice astrocyte culture.

**Table 1:** Clotting activity inhibition from *B. jararacussu* snake venom by cnidarian extracts

	Clotting Time (min.)		
	Samples (1:5)	Samples (1:10)	Samples (1:30)
*B. jararacussu* (20 μg)	1.08	1.10	1.05
*C. gigantea* (body-wall)	1.08	15	16.2
*C. gigantea* (total)	> 3	> 3	12.0
*M. alcicornis*	> 3	1.24	1.13
*S. helianthus*	2.4	> 3	> 3
*P. homomalla*	> 3	> 3	11.4
*B. annulata*	2.1	> 3	> 40

At 3 h after the injection of 50 μg of cnidarian extracts into the gastrocnemius mouse muscle, a slight myotoxic effect from the extracts of *M. alcicornis* and *P. homomalla* was observed, approximately 22% above those observed for the controls injected with PBS alone. The *C. gigantea* (body-wall), *C. gigantea* (total) and *S. helianthus* presented increased activity, while extract of *B. annulata* did not show myotoxic effect.

**Fig. 6:**
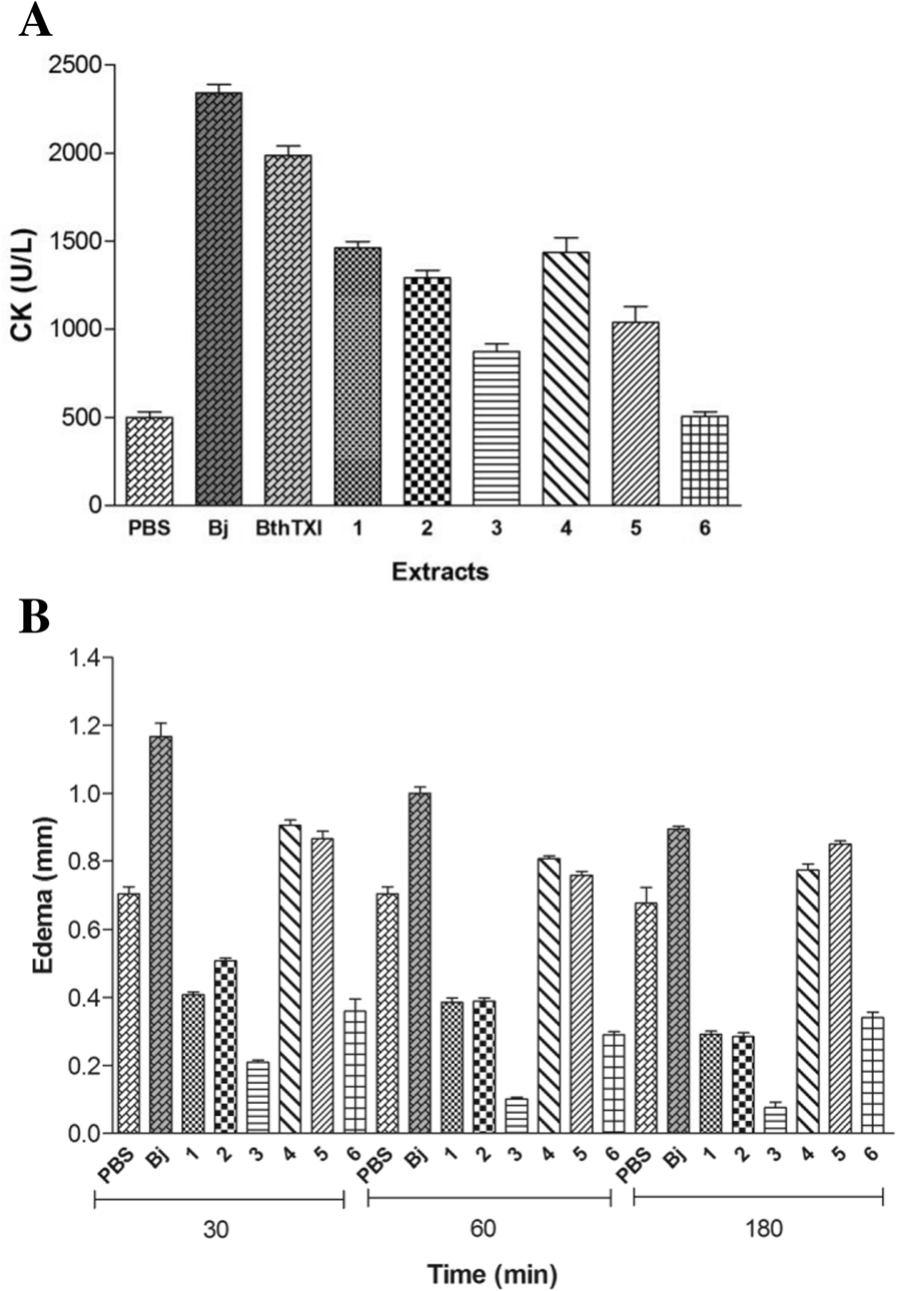
Myotoxic activity and edema-induction by cnidarian extracts*.* (Bj) *B. jararacussu* snake venom and (BthTX-I) Bothropstoxin-I from *B. jararacussu* (1) *C. gigantea* (body-wall), (2) *C. gigantea* (total), (3) *M. alcicornis,* (4) *S. helianthus* (5) *P. homomalla*, (6) *B. annulata.* (**a**) CK activity was measured 3 h after the i.m injection of 50 μL with 50 μg of each cnidarian extracts (1–6) and 20 μg of the Bj and BthTX-I. Results are presented as means ± S.D. (*n* = 4). (**b**) Paw edema in Swiss mice was induced by injection of 100 μg of cnidarian extracts and 10 μg of Bj at 30, 60 and 180 min. Results are reported as mean % ± S. D (*n* = 6)

Some studies have demonstrated the toxic effects of marine animals such as coral *Millepora alcicornis*, which causes systemic reactions in the kidney, lung and liver [8], and *Millepora complanata*, which presents non-peptide toxins highly lethal to mice with LD_50_ of 4.62 μg/g body weight [66]. The other cnidarian extracts tested, *C. gigantea* (body-wall), *C. gigantea* (total) and *S. helianthus*, showed a more pronounced myotoxic effect, smaller only compared with to those induced by *B. jararacussu* venom and the myotoxin BthTX-I, which are highly myotoxic (Fig. 6a). The edema induction was observed only in the presence of extracts from *P. homomalla* and *S. helianthus* (Fig. 6b).

**Fig. 7:**
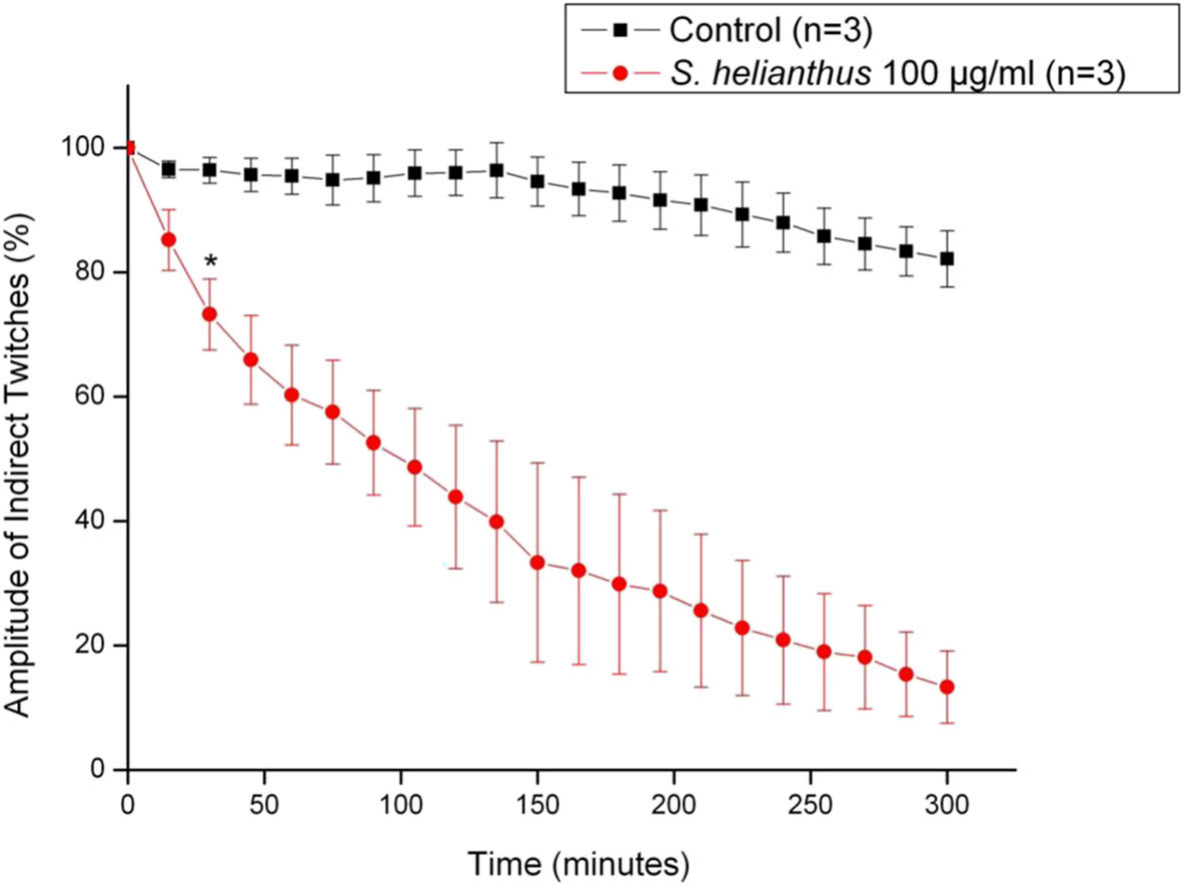
Effects of *S. helianthus* extract (100 μg/mL) upon indirect evoked twitches on mouse phrenic-diaphragm preparation**.** The ordinate represents the % amplitude of twitches relative to the initial amplitude. The abscissa indicates the time (min) after the addition of the extract to the organ bath. Vertical bars represent the SEM; *indicates the point from which there are significant differences relative to control (*p* < 0.05)

**Fig. 8:**
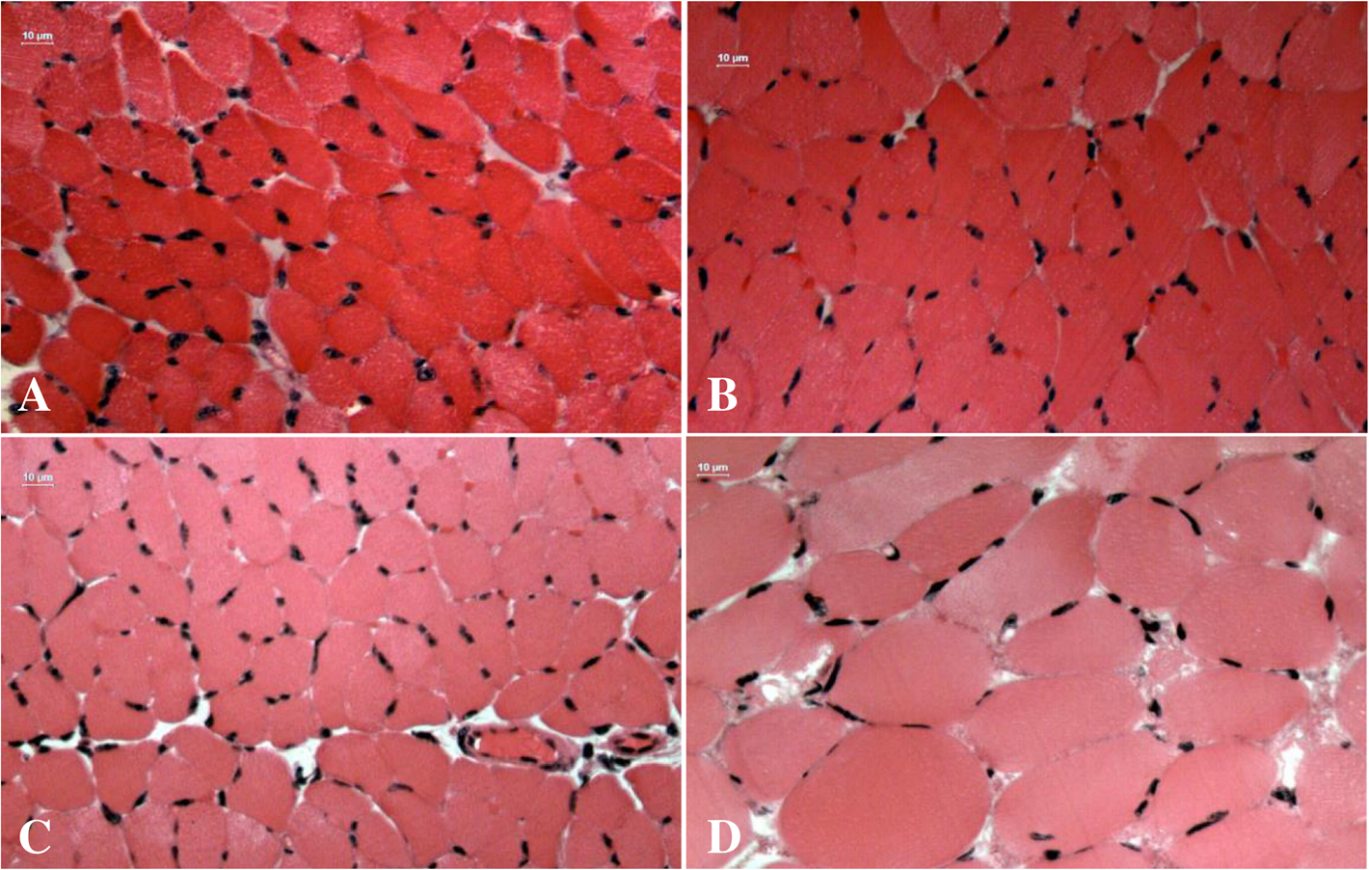
Light micrographs of mouse diaphragm muscles submitted to hematoxylin and eosin staining. Control muscle (**a**) and muscles exposed to extracts of *C. gigantea* (body-wall) (**b**), *C. gigantea* (total) (**c**) and *S. helianthus* (**d**). Note the general normal appearance of fibers with polygonal aspect (f) and endomysium (en). Muscle damage index of (C) and (D) (4.3 ± 1.3, *n* = 5 and 5.5 ± 1.1, *n* = 4, respectively) were not significantly different from that of (A) (2.0 ± 0.4, n = 5). However, muscle damage of B (5.9 ± 0.8, *n* = 5) was significantly higher than that of A. (ANOVA, *p* ≤ 0.05). The remaining cnidarian extracts did not show such activity

In isolated neuromuscular preparation, extract from *S. helianthus* (100 μg/mL) induced a time-dependent blockage of indirect twitches (Fig. 7). After 300 min, the twitch amplitude was reduced by about 84%. In contrast, at the same concentration, extracts from *C. gigantea* (body wall) and *C. gigantea* (total) did not affect the indirectly evoked twitches. Morphological and morphometric analyses revealed an absence of significant damage in diaphragm muscles exposed to *S. helianthus* and *C. gigantea* total extracts (Fig. 8). On the other hand, a slight, but significant level of damage was observed in muscles exposed to *C. gigantea* body wall extract (Fig. 8).

**Fig. 9:**
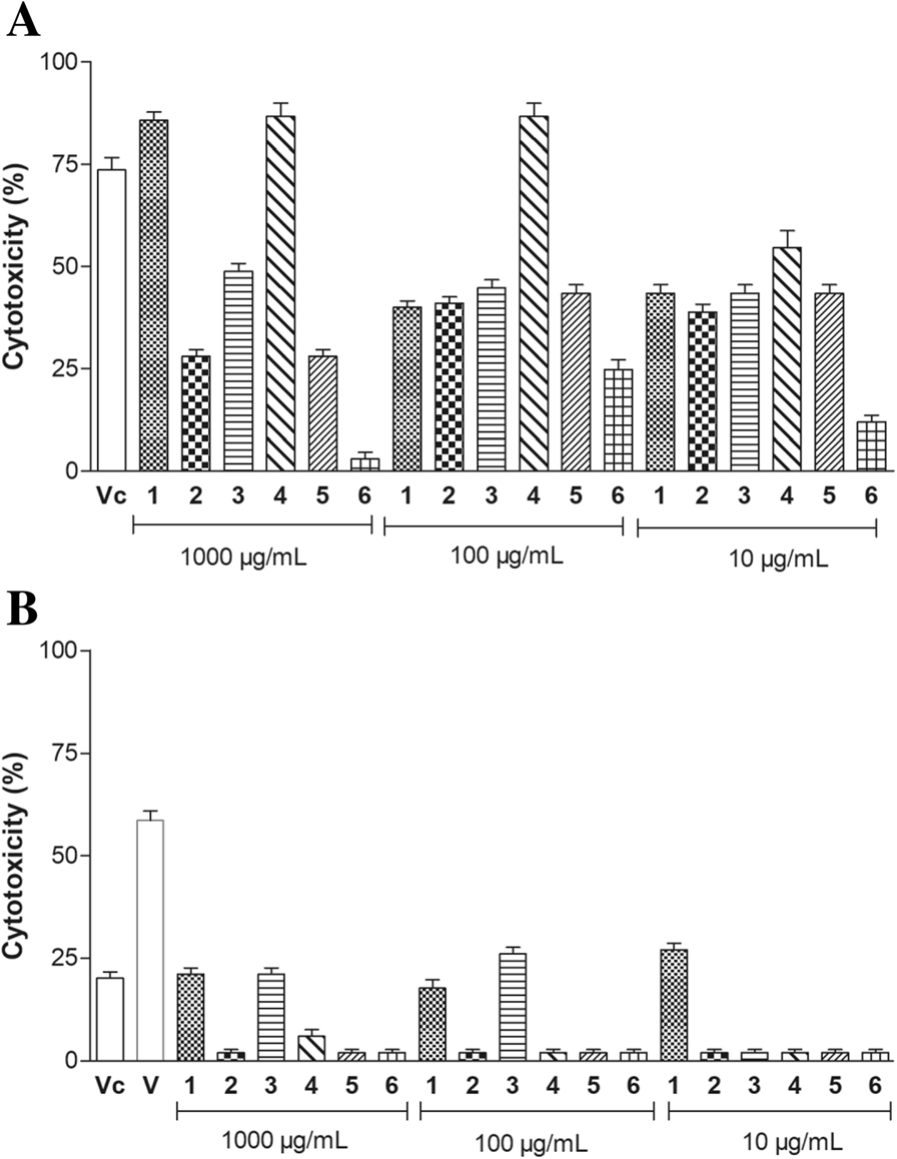
Anti-tumor activity of cnidarian extracts. (V) Vincristine 100 μg/mL, (Vc) Vincristine 10 μg/mL, (1) *C. gigantea* (body-wall), (2) *C. gigantea* (total), (3) *M. alcicornis,* (4) *S. helianthus,* (5) *P. homomalla*, (6) *B. annulata.* (**a**) Antitumoral activity on human acute T-cell leukemia (JURKAT) lines. (**b**) Antitumoral activity on human melanoma (B16F10). Results are presented as means ± SD (*n* = 3)

The cytotoxic activity of cnidarian extracts upon the human tumor cell lines JURKAT (leukemia T) and B16F10 (melanoma) (Fig. 9a and b) showed that all extracts presented anti-tumor activity against JURKAT cells, except the extract *B. annulata*. Additionally, extracts of *C. gigantea* (body-wall) and *S. helianthus* showed significant anti-tumor activity at concentrations of 1000 μg/mL, of which the latter still showed cytotoxic activity of 50% even at the lowest concentration evaluated (10 μg/mL). As to their activity on B16F10 cells, only the extract of *C. gigantea* (body wall) and *M. alcicornis* showed anti-tumor activity at concentrations of 1000 and 100 μg/mL. Some authors proposed that this cytotoxic activity on tumor cell lines is associated with the induction of apoptosis considering the fact that some enzymes isolated from animals exhibit hydrolytic activity by altering the cell membrane [67]. Another study reported the cytotoxic effect of extracts from the marine sponge *Polymastia janeirensis* on a human glioma line (U138MG); in the experiment, both aqueous and organic extracts induced cell death by apoptosis and necrosis [68]. Similar results with extracts from the sponge *Hyattella cribriformis* in ethyl acetate, which exhibited potent growth inhibition of tumor cells as sarcoma, ovarian cancer, colon and breast cell lines [69].

## Conclusions

The neutralization of the clotting induced by *B. jararacussu* snake venom and the inhibition of the hemorrhagic activity induced by *B. moojeni* venom were demonstrated by the majority of the cnidarian extracts tested, whereas the ability to inhibit thrombin-induced coagulation was shown by the *C. gigantea* (body wall). Together with the anti-tumor effect against JURKAT cells demonstrated by all cnidarian extracts tested and the specificity shown against B16F10 cells, these findings constitute important evidence that cnidarians extracts are a rich source of bioactive molecules that should be studied in order to produce data for the development of new alternatives for snakebite envenomation and cancer therapies.
